# Transport metabolons with carbonic anhydrases

**DOI:** 10.3389/fphys.2013.00291

**Published:** 2013-10-10

**Authors:** Joachim W. Deitmer, Holger M. Becker

**Affiliations:** ^1^General Zoology, University of KaiserslauternKaiserslautern, Germany; ^2^Zoology/Membrane Transport, FB Biologie, University of KaiserslauternKaiserslautern, Germany

**Keywords:** acid/base, pH, proton, bicarbonate, lactate

## The concept of the transport metabolon

A metabolon has been defined as a “temporary, structural-functional, supramolecular complex of sequential metabolic enzymes and cellular structural elements, in which metabolites are passed from one active site to another without complete equilibration with the bulk cellular fluids” (Srere, 1985, 1987). This substrate channeling should decrease transit time of intermediates, prevent loss of intermediates by diffusion, protect labile intermediates from solvent, and prevent entrance of intermediates into competing metabolic pathways (Miles et al., 1999). Such metabolons have been found in many metabolic pathways like glycolysis and tricarboxylic acid cycle, as well as in the biosynthesis of DNA, RNA, and proteins (Srere, 1987).

Some of the major acid/base-coupled membrane transporters, such as the Cl^−^/HCO^−^_3_ exchanger AE1 (anion exchanger 1; Band3), the Na^+^/H^+^ exchanger NHE1, the Na^+^/HCO^−^_3_ cotransporter NBCe1, and various monocarboxylate transporters (MCTs) have been demonstrated to physically and/or functionally interact with isoforms of carbonic anhydrase (CA) to form a “transport metabolon.” This functional interaction includes an increase in transport activity, and therefore may enhance both the rate and the capacity of ion/metabolite transport in tissues where these membrane transporters and enzymes are co-localized.

## Interactions requiring catalytic CA activity

First evidence for a transport metabolon, formed between CA and an acid/base transporter, has been presented in 1993 by Kifor et al. (1993) for CAII and AE1. CAII could be co-immunoprecipitated with AE1 when antiserum against the N-terminal of AE1 was used, while immunoprecipitation of AE1 with serum directed against the C-terminal of the transporter failed to co-immunoprecipitate CAII (Vince and Reithmeier, 1998), suggesting that CAII physically binds to the C-terminal tail of AE1. These data were confirmed by affinity blotting and a solid phase binding assay with CAII and a GST fusion protein of the AE1 C-terminal, which showed saturable binding of CAII with a K_1/2_ of 20 nM (Vince and Reithmeier, 1998). Single site mutations identified the acidic cluster L^886^DADD in the C-terminal tail of AE1 as binding site for CAII with at least 2 out of the 3 acidic amino acids being crucial for binding (Vince and Reithmeier, 2000; Vince et al., 2000). This suggests a hydrophobic amino acid followed by at least two acidic amino acids as the CAII binding motif. The amino acid cluster L^886^DADD in the C-terminal of AE1 is located close to the last transmembrane domain. Therefore, binding of CAII to this cluster would position the enzyme close to the transporter pore of AE1 near the inner cell surface. This location may ideally position CAII to hydrate incoming CO_2_ and directly supply the AE1 transporter with a localized substrate pool (Vince and Reithmeier, 2000). Indeed, inhibition of CAII catalytic activity decreased transport activity of AE1 heterologously expressed in HEK293 cells by up to 60% (Sterling et al., 2001).

Reports on a functional interaction between NBCs and carbonic anhydrase go back to the finding that application of the CA inhibitor acetazolamide inhibits transport of bicarbonate across the basolateral cell membrane in renal proximal tubule of rabbits (Burg and Green, 1977; Sasaki and Marumo, 1989; Seki and Frömter, 1992). First evidence for a direct interaction between NBCe1 and CAII was presented by Gross et al. (2002) using isothermal titration calorimetry. CAII was found to bind to the C-terminal peptide of kNBCe1 (amino acids 915-1035) with a K_D_ of 160 ± 10 nM. In analogy to the acidic CAII-binding cluster D^887^ADD found in AE1 (Vince and Reithmeier, 2000), the authors suggested the cluster D^986^NDD within the C-terminal of NBCe1 as the putative CAII binding site. Functional interaction between NBCe1 and CAII was further investigated by heterologous protein expression in *Xenopus* oocytes (Becker and Deitmer, 2007): Both injection and coexpression of CAII increased NBCe1-mediated membrane current and membrane conductance during application of CO_2_/HCO^−^_3_-buffered solution in an ethoxzolamide-sensitive manner. Measurements of intracellular Na^+^ concentrations with ion-selective microelectrodes showed an increase in the rate of NBCe1-mediated Na^+^-flux by two- to four-fold when CAII was injected or co-expressed. CAII-mediated increase in NBCe1 activity as determined by changes in membrane current and slope conductance was dependent on the CAII concentration with a half-maximum enhancement in NBCe1 activity at 20–30 ng CAII/oocyte (Becker and Deitmer, 2007).

Physical and functional interaction with extracellular CAIV could also be demonstrated for the Na^+^/HCO^−^_3_ cotransporter NBCe1 (Alvarez et al., 2003): Transport activity of NBCe1 was determined by fluorometric pH measurements in NBCe1-transfected HEK293 cells subjected to acid loads. Co-transfection of NBCe1 with CAIV significantly increased the rate of NBCe1-mediated pH_i_ recovery. In contrast, CAIV did not increase activity of the NBCe1-mutant G^767^T (positioned in the 4th extracellular loop). In line with the physiological findings, pull-down assays demonstrated physical binding between CAIV and a GST fusion protein of the NBCe1's 4th extracellular loop, but neither to a GST fusion protein of the 4th extracellular loop in which G^767^ was mutated to T, nor to a GST fusion protein of the transporter's 3rd extracellular loop. These data indicate that CAIV can bind to the 4th extracellular loop of NBCe1 [as it binds to the 4th extracellular loop of AE1 (Sterling et al., 2002)] to form the extracellular part of a CAIV-NBCe1 transport metabolon (Alvarez et al., 2003). A transport metabolon of NBCe1 and AE2 with CAIX has recently been suggested also for migrating MDCK cells (Svastova et al., 2012).

It should be noted, however, that the interactions between anion carriers and CAs as described above, have been disputed with respect to the binding and transport activity of the proteins involved (Lu et al., 2006; Piermarini et al., 2007; Yamada et al., 2011). The transport activity was evaluated only by either the current or by the slope conductance in two of these studies, - parameters which may vary in oocytes to a degree which make it difficult to isolate the component contributed by CA, which could be less than 20%. On the other hand, CAII activity may improve substrate supply to bicarbonate transporters even without the requirement for a metabolon involving direct physical interaction, as also pointed out in a recent study on AE1 transport activity (Al-Samir et al., 2013). Therefore, while there is growing support for a functional interaction between bicarbonate transporters and CA activity, the question whether this interaction requires direct binding of the proteins involved remains not finally settled at this point.

## Interactions independent of catalytic CA activity

Lactate, pyruvate, and ketone bodies are transported into and out of cells via monocarboxylate transporters (MCT, SLC16), of which 14 isoforms have been described. The first four of these 14 isoforms (MCT1-4) have been shown to transport monocarboxylates together with H^+^ in a 1:1 stoichiometry (Carpenter and Halestrap, 1994; Bröer et al., 1998). All MCTs have a classical 12 transmembrane-helix structure, with both the C- and N-terminal located intracellularly (Halestrap and Price, 1999). Trafficking, but also regulation of transport activity of MCT1-4 is mediated by the ancillary proteins basigin (CD147) or embigin (gp70), which bind to the transporter (Wilson et al., 2005).

First evidence that the non-catalytic interaction between MCT and CAII depends on a direct interaction between the two proteins was shown by injection of CAII that was bound to an antibody prior to the injection. In this experiment CAII was not able to enhance transport activity of MCT1 in *Xenopus* oocytes, suggesting a steric suppression of the interaction by the antibody (Becker et al., 2005). In the same study, truncation of the MCT1 C-terminal tail (MCT1-D56) led to loss of interaction between MCT1 and CAII in *Xenopus* oocytes.

By introduction of single site mutations in the C-terminal of MCT1 and subsequent expression of these mutants in CAII-injected *Xenopus* oocytes, the two glutamate residues E^489^ and E^491^ flanking the acidic cluster E^489^EE within the MCT1 C-terminal tail could be identified to be crucial for the functional interaction with CAII (Stridh et al., 2012). Direct binding between CAII and the MCT1 C-terminal tail was shown by co-immunoprecipitation when the acidic cluster E^489^EE was intact, while mutation of E^489^ and/or E^491^ suppressed the binding between MCT1-CT and CAII. This suggests that cytosolic CAII can bind to the C-terminal tail of MCT1, which presumably positions the enzyme close enough to the pore of the transporter for efficient H^+^ shuttling.

It has been demonstrated that the enhancing effect of CAII on H^+^/lactate influx via MCT1 and MCT4 increased with increasing extracellular proton concentration, but decreased with extracellular lactate concentration (Becker and Deitmer, 2008; Almquist et al., 2010; Becker et al., 2010). This gave rise to the hypothesis that CAII-induced augmentation of MCT transport activity is linked to the H^+^ gradient across the cell membrane. Mathematical modeling of the transport mechanism suggested that CAII increases the rate constant for the binding and the release of H^+^ at the transporter by providing additional H^+^ binding sites, and thereby speeds up lactate/proton co-transport (Almquist et al., 2010).

CAII facilitates H^+^ transfer between the zinc-bound water and the solvent surrounding the enzyme by the side chain of H64, which shuttles H^+^ between the bulk solvent and a network of well-ordered hydrogen-bonded water molecules in the enzyme's active-site cavity (Fisher et al., 2007). Co-expression of the mutant CAII-H64A, lacking the intramolecular H^+^ shuttle, with MCT1 or MCT4 in *Xenopus* oocytes resulted in no increase in transport activity (Becker et al., 2011). This led to the notion that CAII may provide or subtract protons to or from the transporter, respectively, via its intramolecular H^+^ shuttle. Injection of 4-methylimidazole (4-MI), which acts as a H^+^ donor/acceptor, indeed restored the ability of the CAII-H64A mutant to enhance transport activity of MCT1/4 (Becker et al., 2011). It is hypothesized that 4-MI binds at various positions within the active site cavity of CAII, which would then rescue proton shuttling in the enzyme (Duda et al., 2001).

While injection of CAII does not increase transport activity of MCT2, heterologously expressed in *Xenopus* oocytes, co-expression of the extracellular isoform CAIV led to a doubling in the rate of lactate-induced acidification (Klier et al., 2011). As already observed for MCT1/4 and CAII, the interaction between MCT2 and CAIV persisted in the nominal absence of CO_2_/HCO^−^_3_ and was insensitive to inhibition of the enzyme's catalytic activity. The non-catalytic nature of the interaction was confirmed by co-expression of MCT2 with the catalytically inactive mutant CAIV-V165Y, which increased MCT2 activity as did CAIV-WT. Interestingly, removing the intramolecular H^+^-shuttle (CAIV-H88A), analogous to the proton shuttle in CAII, which had been shown to be crucial for the interaction of CAII with MCT1/4, led to a significantly smaller increase in MCT2 activity than did CAIV-WT, but did not fully abolish the interaction with MCT2 (Klier et al., 2011). It has been suggested that this residual enhancement could be due to a second, not yet identified, proton shuttle in CAIV (Hurt et al., 1997; Klier et al., 2011). Furthermore, CAIV could only increase activity of MCT2 when the transporter was co-expressed with its trafficking protein embigin (gp70). From this it was concluded that the interaction between MCT2 and CAIV may not depend on a direct binding between MCT2 and CAIV, as suggested for MCT1/4 and CAII, but may be mediated by binding of CAIV to embigin, which could act as a mediator for CAIV-induced transport enhancement of MCT2 (Klier et al., 2011). As both N- and C-terminal tails of MCT2 are located intracellularly, and the transporters' short extracellular loops might not provide enough space for binding of CAIV, the two extracellular globular domains of embigin may well provide a binding site for CAIV.

## Conclusions and perspectives

Transport metabolons between carbonic anhydrases and acid/base-coupled membrane transporters display a great versatility. Not only that most of the H^+^ and HCO^−^_3_ carrying transporters interact with one or several isoforms of carbonic anhydrase, the mode of interaction also shows some unexpected variability. Membrane transporters may interact with intracellular and extracellular carbonic anhydrases, and this interaction may be direct including physical binding, or may possibly be mediated by chaperones associated with the transport proteins. Most of the interactions require the catalytic activity of the carbonic anhydrases, and functional interaction may be attributable mainly to the faster conversion of CO_2_, HCO^−^_3_, and H^+^ by CAs, while co-localization and perhaps physical attachment between acid/base-coupled transporters and CAs may meet the structural and spatial requirements of this process. The isoform-specific interplay between all monocarboxylate transporters and carbonic anhydrase isoforms so far tested, is *in*dependent of the anhydrase enzymatic activity. Moreover, similar binding domains have been identified in various MCT and CA isoforms. Future studies on the interaction between transporters and carbonic anhydrases, should help to unravel the molecular mechanisms of this functional metabolon system in more detail, from which we may learn more about transport metabolons in general.
